# Regulating Toxin-Antitoxin Expression: Controlled Detonation of Intracellular Molecular Timebombs

**DOI:** 10.3390/toxins6010337

**Published:** 2014-01-15

**Authors:** Finbarr Hayes, Barbara Kędzierska

**Affiliations:** 1 Faculty of Life Sciences and Manchester Institute of Biotechnology, The University of Manchester, 131 Princess Street, Manchester M1 7DN, UK; 2 Department of Molecular Biology, University of Gdańsk, Wita Stwosza 59, Gdańsk 80-308, Poland

**Keywords:** bacteria, toxin-antitoxin, transcription, regulation, antibacterial

## Abstract

Genes for toxin-antitoxin (TA) complexes are widely disseminated in bacteria, including in pathogenic and antibiotic resistant species. The toxins are liberated from association with the cognate antitoxins by certain physiological triggers to impair vital cellular functions. TAs also are implicated in antibiotic persistence, biofilm formation, and bacteriophage resistance. Among the ever increasing number of TA modules that have been identified, the most numerous are complexes in which both toxin and antitoxin are proteins. Transcriptional autoregulation of the operons encoding these complexes is key to ensuring balanced TA production and to prevent inadvertent toxin release. Control typically is exerted by binding of the antitoxin to regulatory sequences upstream of the operons. The toxin protein commonly works as a transcriptional corepressor that remodels and stabilizes the antitoxin. However, there are notable exceptions to this paradigm. Moreover, it is becoming clear that TA complexes often form one strand in an interconnected web of stress responses suggesting that their transcriptional regulation may prove to be more intricate than currently understood. Furthermore, interference with TA gene transcriptional autoregulation holds considerable promise as a novel antibacterial strategy: artificial release of the toxin factor using designer drugs is a potential approach to induce bacterial suicide from within.

## 1. Introduction: A Brief Overview of Toxin-Antitoxin Complexes

Toxin-antitoxin (TA) systems are compact modules, usually comprising a pair of genes coding for a toxin and a cognate antidote. TA complexes are abundant on plasmids and chromosomes of many bacterial and archaeal species [1,2,3,4,5,6]. The toxic components of TA systems can be regarded as intracellular molecular bombs whose release from a complex with their cognate antitoxins triggers bacterial cell death or stasis. In this review we provide current insights into the mechanisms by which expression and activation of these modules are controlled at the transcriptional level which is crucial to understand TA functioning and their possible practical exploitation as emerging targets for novel antibacterial agents.

Plasmid encoded TA systems act in postsegregational killing of bacterial cells that have failed to inherit a plasmid during cell division [7]. In progeny deprived of a plasmid, proteolytic degradation of the more labile antitoxin and the lack of its *de novo* synthesis lead to the release of the stable toxin which interacts with its intracellular target, causing cell death or inhibition of metabolic processes. Thus, bacteria become “addicted” to TA modules located on plasmids, as daughter cells die when the plasmid is lost. Chromosomal TAs instead are involved in response to various stress conditions, ensure genomic stability, function as anti-addiction modules, or may act only as selfish genetic entities [3,8,9,10]. TAs also are implicated in antibiotic persistence, biofilm formation, and bacteriophage resistance [11,12,13].

TA cassettes currently are classified into five types, based on the characteristics of the antitoxins and the mechanisms by which they counteract the cognate toxins [14,15]. In a typical type I TA system the toxin is a small hydrophobic protein whereas the antitoxin acts as an antisense RNA which pairs with the toxin mRNA [16,17]. Inhibition of toxin translation occurs via degradation of RNA duplexes or by masking of the ribosome binding site [18]. In type II modules both toxin and antitoxin are small proteins which form a stable complex. The antitoxin blocks activity of the toxin by hiding the region responsible for toxicity [3]. In type III complexes an antitoxin RNA interacts directly with the toxin protein and in this way abolishes its toxicity [19]. Type IV consists of a protein toxin and a protein antitoxin, the latter preventing toxin to access its target [20,21]. Finally, type V TAs comprise a protein antitoxin which acts as a ribonuclease that specifically cleaves the toxin mRNA and disables its synthesis [22]. TA systems belonging to the first two types are the most abundant in the prokaryotic world, whereas only single representatives are known to date for the other three classes.

**Figure 1:**
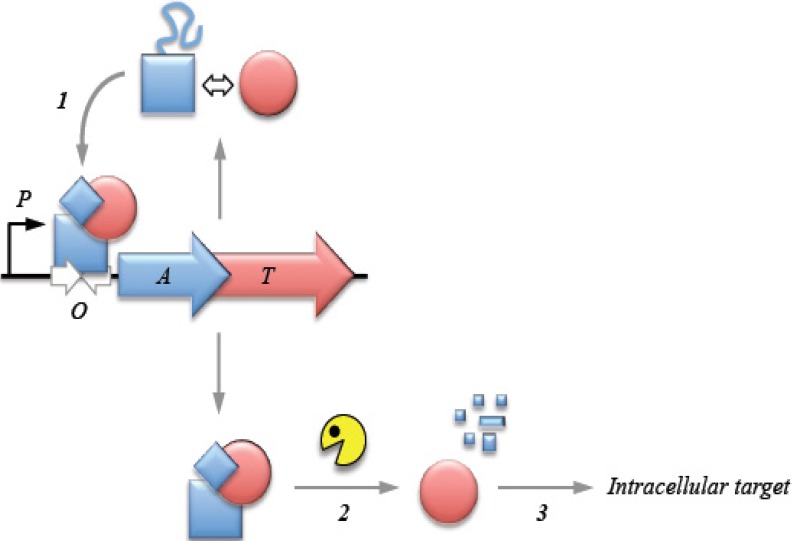
Action of a generic type II toxin-antitoxin (TA) complex in which both factors are proteins. The antitoxin (A) and toxin (T) genes are expressed in an operon. A poorly structured domain within the antitoxin protein is remodelled by the toxin to produce a stable complex that autoregulates operon expression at the transcriptional level (**1**); In response to certain environmental conditions, e.g., nutrient limitation or antibiotic exposure, the antitoxin is proteolytically degraded (**2**); The toxin is thereby released to act on its intracellular target to induce cell dormancy or stasis (**3**). *P*, promoter; *O*, operator.

This mini review focuses on type II TA modules. The action of a generic type II complex in which both factors are proteins is presented in Figure 1. The toxin and antitoxin factors associate tightly to form the complex. Genes for both proteins are co-expressed from a promoter that is negatively autoregulated by the TA complex. In response to certain environmental conditions, including nutrient limitation or antibiotic exposure, the antitoxin undergoes Clp or Lon protease mediated degradation. The toxin is thereby released and can reach its intracellular target to induce cell dormancy, stasis or death [3]. Toxins hinder cellular activities by targeting various structures and key molecular processes [6,14]. Most class II toxins examined to date regulate the translation process by acting as endoribonucleases [23,24]. Some of these endoribonucleases cleave free mRNA in a sequence dependent manner, whereas others target mRNA associated with the ribosomal A site. Certain type II toxins instead inhibit the translation machinery by cleaving initiator tRNA, by phosphorylation of the elongation factor EF-Tu, or by binding to ribosomal subunits [6,25,26,27]. In contrast with factors that target the translation process, certain type II toxins affect DNA replication by direct inhibition of gyrase activity [28,29,30] or by interfering with the β sliding clamp [31]. There are also known examples of cell wall synthesis inhibitors that act by phosphorylating a peptidoglycan precursor [32].

## 2. Transcriptional Autoregulation Is a Characteristic Feature of Type II TA Cassettes

As summarized above, type II complexes comprise a toxin and antitoxin that both are proteins [7]. Toxicity is neutralized by physical association of the two factors. When released from the complex, the toxin targets a specific intracellular component to trigger temporary stasis or cell death. In view of its latent potential to interfere with vital cellular processes, inadvertent release of the toxin is catastrophic for cell viability. To circumvent this possibility, transcriptional autoregulation is a characteristic feature of TA cassettes that ensures balanced toxin and antitoxin production.

Transcriptional autoregulation of type II modules typically is mediated by the antitoxin that partially represses expression by binding to an operator site which overlaps the promoter motifs recognized by RNA polymerase. However, full repression is achieved only by binding of the TA complex to the site. Type II antitoxins usually comprise a well-structured N-terminal domain that mediates DNA binding and a partly-structured C-terminal region. The toxin interacts with and restructures the latter thereby stabilizing the antitoxin for more effective operator binding (Figure 1). The following sections outline salient features of transcriptional autoregulation in selected type II TA complexes that illustrate the variations on a theme that have developed in TA gene expression control.

## 3. CcdA-CcdB: Transcriptional Repression of a DNA Gyrase Poison

The CcdA-CcdB TA complex first was identified as a maintenance system encoded by the F plasmid in *Escherichia coli* [33]. Genes for homologous complexes are distributed widely on both bacterial plasmids and chromosomes, although are less common than certain other TA loci [34]. DNA gyrase is an essential type II topoisomerase that introduces negative supercoils into bacterial DNA. The CcdB toxin poisons covalent gyrase-DNA complexes both by entrapping the gyrase cleavage complex and by inhibiting the enzyme’s catalytic reactions [28,35]. The CcdA antitoxin sequesters CcdB thereby blocking interference with gyrase [36].

**Figure 2:**
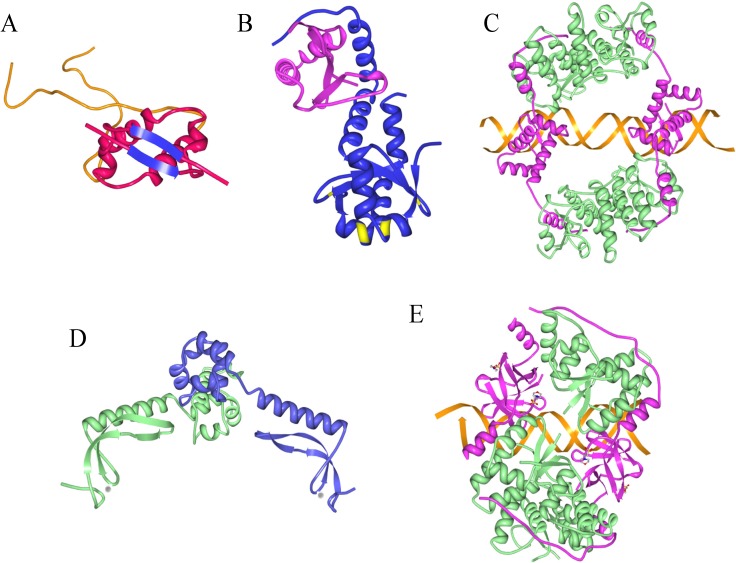
Tertiary structures of selected antitoxins and TA complexes. (**A**) solution structure of CcdA antitoxin [44]. The structure comprises a dimeric, N-terminal ribbon-helix-helix (RHH) fold (red and blue) that binds DNA connected to a pair of C-terminal extensions (orange) that become structured upon interaction with the CcdB toxin [44]. The antiparallel β-strands in the RHH fold that insert into the DNA major groove are highlighted in blue. (**B**) crystal structure of the YefM-YoeB complex [45]. The YefM antitoxin dimer and YoeB toxin monomer are coloured in blue and magenta, respectively. Conserved arginine residues in the N-terminal segment of YefM that are involved in operator recognition [46] are highlighted in yellow. (**C**) crystal structure of the FitA-FitB-DNA complex [47]. FitA and FitB are shown in magenta and green, respectively. The structure comprises four FitA-FitB heterodimers assembled on a 36-bp DNA segment (orange) from the regulatory region usptream of *fitA-fitB*. (**D**) crystal structure of the MsqA antitoxin [48]. Monomers within the dimeric structure are coloured green and blue. Zinc ions involved in maintaining MsqA structural stability are shown as red spheres. (**E**) crystal structure of the VapB2-VapC2 complex of *Rickettsia felis* [49]. The structure consists of four VapC toxin molecules (green) and four antitoxin monomers (magenta) which form a tetramer of heterodimers assembled on a 26-bp DNA segment. Images constructed using the Research Collaboratory for Structural Bioinformatics Protein Data Bank and the Molecular Biology Toolkit [50].

**Figure 3:**
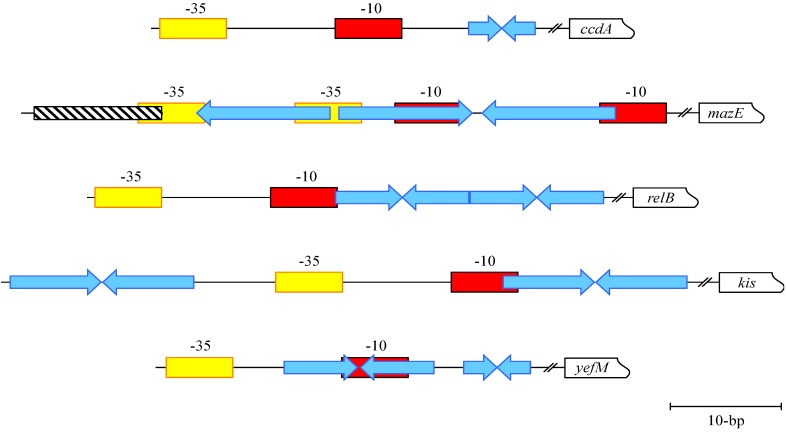
Organization of the promoter-operator regions upstream of the *ccdA-ccdB*, *mazE-mazF*, *relB-relE*, *kis-kid*, and *yefM-yoeB* TA genes (top to bottom). −10 and −35 promoter motifs are coloured red and yellow, respectively. Repeat sequences are denoted by blue arrows; the sequences of these repeats differ among the different systems. The FIS binding site in the *mazE-mazF* promoter region is shown as a hatched box.

The *ccdA-ccdB* genes form an operon that is autoregulated at the transcriptional level. Although CcdA alone binds the *ccdA-ccdB* promoter-operator region *in vitro*, repression *in vivo* is mediated by the CcdA-CcdB complex [37,38,39,40]. The two-protein complex also binds DNA more avidly and with higher specificity *in vitro* than does CcdA alone [41,42]. CcdB does not bind DNA [37]. The N-terminal region of CcdA adopts a dimeric, ribbon-helix-helix (RHH) fold that is characteristic of a broad family of prokaryotic transcriptional repressors that regulate a variety of cellular processes (Figure 2A) [43]. Like other RHH proteins, the CcdA antitoxin binds DNA by insertion of a pair of positively charged, antiparallel β-strands into the major groove. The C-terminal region of CcdA is highly mobile but is stabilized by interaction with CcdB which in part may be due to obscuring of the site recognized on CcdA by the Lon protease [44]. CcdA recognizes a 6-bp palindrome downstream of the -10 promoter element for the *ccdA-ccdB* operon (Figure 3). This hexamer is thought to be a nucleation site for assembly of an extended superstructure in which multiple CcdA-CcdB complexes spiral around ~120-bp of DNA that encompasses the promoter-operator and the 5’ end of the *ccdA* gene [39,40,41,44]. The biological purpose of this higher order nucleoprotein complex is uncertain, but may permit a gradated transition of *ccdA-ccdB* expression dependent on the extent of spreading of the CcdA-CcdB complex from the nucleation point.

## 4. MazE-MazF: Autoregulation of a Chromosomally Encoded Type II Paradigm

The MazF toxin encoded by *E. coli* is the archetype of a broad family of sequence-specific endoribonucleases that cleave either free mRNA or ribosome-associated transcripts resulting in translation inhibition [23,24]. The activity of MazF is counteracted directly by the MazE antitoxin [51,52]. Conflicting evidence indicates that the MazE-MazF complex either is a primitive programmed cell death mechanism in bacteria, or that MazF toxicity instead induces reversible bacteriostasis from which cells can recover by subsequent production of MazE antitoxin as discussed elsewhere [3,9,53,54].

Antibiotics that inhibit transcription and/or translation prevent replenishment of the MazE antitoxin. As a consequence MazF, which is less proteolytically labile than MazE, is released to cause toxicity. Stressful conditions such as extreme amino acid starvation also inhibit *mazEF* transcription. When uncharged tRNA species occupy the ribosomal A-site during amino acid starvation, the RelA protein is activated and catalyzes synthesis of the alarmone guanosine tetraphosphate (ppGpp). Overproduction of ppGpp represses the *mazEF* cassette by interfering with RNA polymerase function. The consequent decrease in MazE levels liberates the MazF toxin to act intracellularly [51]. Interestingly, the *mazG* gene that is cotranscribed with *mazEF* provides another level of control to MazF action. MazG is a nucleoside triphosphate pyrophosphohydrolase that hydrolyzes dNTPs, thereby depleting ppGpp concentration. MazG activity is counteracted by the MazE-MazF complex. However, when amino acid starvation induces elevated ppGpp concentration with a concomitant reduction in expression of the *mazEFG* module, MazG function is no longer blocked and ppGpp level decreases. Thus, the action of the MazE-MazF complex is balanced by the antagonistic activities of ppGpp that blocks *mazEFG* transcription and by MazG which hydrolyzes the alarmone [55].

The *mazEFG* cassette is preceded by two promoters whose relative strengths differ approximately ten-fold [51,56]. Transcription of the genes is autorepressed: MazE partially regulates both promoters but stronger repression is achieved by the MazE-MazF complex. Repression involves binding of the complex to an operator that comprises three related, adjacent repeat motifs that overlap the promoter region (Figure 3). By interacting with the first and second motifs or the second and third motifs, MazE-MazF may autorepress expression either to varying levels and/or in response to different physiological stimuli [56]. The MazE protein forms a homodimer within the MazE-MazF crystal structure, adopting a swapped-hairpin β-barrel structure with two extended C-terminal regions that interact with MazF. This structure identifies MazE as a member of the superfamily of DNA binding proteins typified by the transcription factor AbrB. Operator recognition by MazE is thought to involve basic residues on the underside of the β-barrel element [52].

Interestingly, the factor for inversion stimulation (Fis) also binds to the *mazEFG* promoter region (Figure 3) and modestly up-regulates *mazEFG* expression [56]. Fis is a small, nucleoid-associated protein that binds to sites throughout bacterial chromosomes thereby regulating a wide array of genes, both directly by promoter interaction and indirectly by altering DNA structure during various growth stages [57,58]. Fis abundance varies dramatically during cell cycle progression [59]. Correspondingly, the expression of the *mazEFG* operon may be modulated by fluctuating Fis levels [56]. More broadly, the *mazEFG* genes form one component in an interconnected set of responses to DNA damage and other stresses, and also sense population density [53,60,61], suggesting that their transcriptional regulation may prove to be more intricate than currently understood. Intriguingly, Fis also influences expression of the *vapBC-1* TA cassette in nontypeable *Haemophilus influenzae* [62] (see below) suggesting that Fis and other global transcriptional regulators may prove to have a wide role in TA regulation.

## 5. Phd-Doc, RelB-RelE and Kis-Kid: Transcriptional Regulation and Conditional Cooperativity

The lysogenic form of bacteriophage P1 exists as a low copy number plasmid that is stabilized by the Phd-Doc type II TA. The Doc toxin possesses high and low affinity binding sites for the Phd antitoxin. The Phd-Doc complex is a highly effective transcriptional repressor when both sites are occupied, whereas Phd alone represses only weakly [63,64]. If the Doc:Phd ratio increases sufficiently, the antitoxin molecules are captured preferentially by the high affinity binding sites in the excess Doc proteins. The consequent alteration in quaternary structure of the Phd-Doc complex disrupts the repressive complex thereby inhibiting operator site binding. This leads to derepression of the *phd-doc* operon specifically when toxin levels are elevated. More Phd is produced as a consequence to allow both trapping of excess Doc and re-establishment of transcriptional repression [64]. This phenomenon in which TA operon expression is modulated by the toxin:antitoxin ratio is termed *conditional cooperativity* [65,66] and reflects earlier observations that excess toxin often perturbed operator binding by TA complexes [37,44,67,68,69]. Conditional cooperativity is thought to calibrate TA levels so that random induction of operon expression is curtailed and also to permit efficient sequestration of excess toxin so that toxin-induced temporary stasis can be reversed [70]. 

RelB-RelE is one of the most prevalent and well-studied TA complexes [71,72]. Like MazF, the RelE toxin is an endoribonuclease, albeit with a different cleavage specificity on mRNA [73,74,75]. Moreover, unlike MazF, transcript cleavage by RelE is translation-dependent [74]. The N-terminus of the RelB antitoxin adopts a dimeric RHH fold connected to a pair of C-terminal extensions that capture two RelE monomers, forming an open V-shaped heterotetrameric stucture in which RelB is positioned at the vertex [65,76,77]. This configuration blocks access by the toxin to the ribosomal A site and induces structural rearrangements that perturb the endoribonuclease pocket [65,78].

Transcription of the *relB-relE* module is autoregulated in *E. coli*. In common with numerous TA complexes, the RelB antitoxin represses transcription weakly whereas the RelE toxin is a co-repressor [79]. The operator site recognized by RelB-RelE comprises a pair of tandem 12-bp inverted repeats one of which overlaps the −10 promoter motif for the *relBE* module (Figure 3) [76,77]. The RelB-RelE complex exhibits conditional cooperativity: RelE promotes operator binding by RelB at subequimolar ratios but ablates binding at higher concentrations [66]. In agreement, modelling of the complex onto DNA suggests that a RelB dimer associated with a single RelE monomer binds one inverted repeat within the operator. The second inverted repeat is occupied by another heterotrimeric complex that interacts cooperatively with the adjacent complex. In contrast, steric clashes between RelE molecules are predicted to block operator binding by a complex with an equimolar RelB:RelE ratio [65]. The outcome of conditional cooperativity in the RelB-RelE system parallels that of the Phd-Doc complex described above [64], *i.e.*, differential regulation of the cognate TA genes by toxin:antitoxin ratios. As with Phd-Doc, RelE possesses high and low affinity binding sites for RelB that likely influence the RelB:RelE ratio intracellularly. The low affinity site for the RelB-RelE interaction could play a role in the interaction of excess of toxin with the TA repressor bound to the operator and in the release of the repressor under these circumstances. However, unlike with Phd-Doc, these sites are not invoked in conditional cooperativity. Instead, interactions between RelB dimers positioned on the inverted repeats and stabilized by subequimolar binding of RelE are sufficient for cooperative operator recognition [65].

The Kis-Kid type II TA complex is encoded by the R1 plasmid of *E. coli*. Kid is a sequence-specific, translation-independent endoribonuclease whose activation inhibits the growth of plasmid-free cells [80]. The protein is a member of a broad group of structurally-related toxins that includes MazF and CcdB [1], whereas the Kis antitoxin is homologous to MazE [81]. The *kis-kid* operon is transcriptionally autoregulated. As is characteristic of type II complexes, the antitoxin partially represses expression but the Kis-Kid complex represses more effectively [82]. The operator site recognized by Kis-Kid comprises a pair of imperfect 18-bp inverted repeats that are separated by a 33-bp spacer in the promoter region for the *kis-kid* genes (Figure 3). The repeat that overlaps the −10 promoter box is bound more strongly by the complex suggesting that the interaction of Kis-Kid with RNA polymerase modulates *kis-kid* expression [69]. Kis and Kid assemble into numerous complexes *in vitro* [81]. As is characteristic of conditional cooperativity, complexes that contain equimolar or subequimolar Kis:Kid concentrations bind the operator site most effectively. In contrast, less stable complexes with DNA are formed when the Kid toxin is in excess of Kis antitoxin and transcriptional repression may be limited. Consequent *kis-kid* expression will rebalance the Kis:Kid ratio away from toxin excess [69]. Two transcripts are produced from the *kis-kid* operon in equimolar concentrations and with similar half-lives. The longer of these transcripts spans both genes and is also processed by limited degradation to a shorter species that terminates within *kid*. Moreover, translation of the Kid toxin and Kis antitoxin are coupled which further minimizes the synthesis of Kid in the event that insufficient Kis has been translated [83]. Transcriptional activity of the *kis-kid* operon is also dependent on plasmid copy number [84]. Thus, conditional cooperativity, RNA processing and translational coupling, act in concert to ensure balanced production of the Kis antitoxin relative to the Kid toxin. However, conditional cooperativity is not a universal control mechanism among type II TA systems [85] and is predicted to be one of a variety of emerging regulatory strategies that have evolved to fine-tune TA gene expression.

## 6. YefM-YoeB and Axe-Txe: Diverse Transcriptional Control of Homologous TA Complexes

The YefM-YoeB system of *E. coli* is a hybrid type II TA complex: the YoeB toxin and YefM antitoxin are related evolutionarily to RelE and Phd, respectively, whereas the usual associations are RelB-RelE and Phd-Doc [86,87,88]. YoeB binding to the 50S ribosomal subunit prevents the formation of the initiation complex and induces mRNA cleavage three bases downstream of the initiation codon [89]. However, the toxin’s catalytic fold is concealed when it is bound to YefM thereby blocking its enzymatic activity. This interaction, which also stabilizes YefM, is crucial for ensuring that free YoeB is not available erroneously to degrade mRNA [45].

The *yefM-yoeB* genes are transcriptionally autoregulated. YefM is the principal repressor, whereas YoeB is a repression enhancer [90]. YefM lacks a canonical DNA binding motif [45], but dual conserved arginine residues in the N-terminal segment of the protein are involved in operator recognition (Figure 2B) [46]. Free YefM sequentially recognizes adjacent long and short DNA palindromes during transcriptional repression of the *yefM-yoeB* operon. The palindromes share core hexamer 5’-TGTACA-3’ motifs, possess a centre-to-centre distance of 12-bp and overlap the *yefM-yoeB* promoter (Figure 3). The YefM-YoeB complex binds the palindromes more avidly than free antitoxin via cooperative interactions [46,90]. Changing the inter-palindrome spacing perturbs cooperative binding to the repeats: YefM-YoeB interaction with the long repeat is retained but binding to the short repeat is disrupted [46]. Paired hexamer motifs are frequent in *yefM-yoeB* regulatory regions in diverse genomes suggesting that interaction of YefM-YoeB with these motifs is a conserved mode of operon transcriptional autoregulation [90].

The Axe-Txe complex encoded widely by enterococcal plasmids is homologous to YefM-YoeB [87]. Analogously, the Axe antitoxin represses the *axe-txe* promoter weakly whereas Axe-Txe represses more strongly [91]. However, an internal promoter within *axe* also directs expression of the downstream *txe* toxin gene. This internal promoter is not regulated by Axe-Txe and is essential for the function of the complex as a plasmid maintenance system suggesting that it plays a vital role in setting the Axe:Txe ratio. A cryptic transcript that originates within *txe* and a putative transcription terminator in the region 3’ of the operon may be additional regulatory elements that contribute to transcriptional control of *axe-txe* [91]. The *yefM-yoeB* operon of *Streptococcus pneumoniae* provides another example of multilayered transcriptional control. In this case, a pair of promoters, one of which is constitutive and one of which is autoregulated by YefM-YoeB, are located upstream of the genes [92].

The interactions between the homologous YefM-YoeB and Axe-Txe complexes are species-specific. Accordingly, the complexes repress expression of the cognate promoters, but not of the non-cognate promoters, even though both operator regions are composed of a pair of inverted repeats with the same hexameric core [88]. However, a single substitution near the C-terminus which converts a Txe-specific residue to a YoeB-specific amino acid permitted neutralization of Txe by YefM *in vivo*. Moreover, the complex of wild-type YefM and mutated Txe partially corepressed the *yefM-yoeB* promoter [88]. These data illustrate that subtle amino acid sequence changes can impose interaction specificity, including transcriptional regulation specificity, on homologous TA complexes, and provide insight into the mechanisms by which expression of multiple, homologous type II TA complexes in a single host may be regulated differentially under disparate physiological conditions [93].

## 7. Diverging from the Canonical Pattern of Type II TA Transcriptional Control: Tripartite Protein Complexes and a Toxin that Directly Binds DNA

Certain exceptions to the general pattern of type II TA transcriptional regulation have been described. Three component TA modules include the *pasABC* complex encoded by plasmid pTF-FC2, *paaA-parE-paaR* of *E. coli* O157:H7, and the *ε-ζ-ω* system specified by streptococcal plasmid pSM19035. 

The *pasA* gene codes for an antitoxin and *pasB* encodes a toxin [94]. The *pasABC* promoter undergoes full autorepression by the PasAB complex, and the PasC protein has little effect on promoter expression [95]. Instead, PasC enhances the neutralizing effect of the antidote [94].

In the case of the *paaR-paaA-parE* operon, the PaaA antitoxin and ParE toxin form a complex that autorepresses the main promoter only partially. The transcriptional regulator encoded by an upstream gene within the same operon, PaaR, is required for full down-regulation of transcription. These different repressor complexes probably act independently [96]. The PaaR repressor also is essential in maintaining an appropriate PaaA:ParE ratio [96]. 

Another tripartite type II TA system is ε-ζ-ω which was discovered initially on plasmid pSM19035 of *Streptococcus pyogenes*. In this case the ζ toxin and ε antitoxin have no roles in transcriptional control. Instead, their expression is inhibited solely by the ω protein encoded by the first gene in the operon. In addition to being a repressor of its own promoter, ω is encoded widely by plasmids belonging to the Inc18 family and acts as a global negative regulator that also controls transcription of genes required for plasmid copy number control and stable inheritance, thereby promoting accurate plasmid segregation [97,98,99,100]. Like certain other type II TA transcriptional regulators, including CcdA (Figure 2A) and RelB, ω is a RHH protein [101]. However, the binding site recognized by ω is distinctive, comprising palindromic and non-palindromic DNA heptad repeats (5’-NATCACN-3’) in the cognate operator sites [98].

The VapC subfamily of PIN domain proteins forms the toxin component of a widespread type II TA complex. PIN domain proteins cleave single-stranded RNA and are characterized by a cluster of strictly conserved acidic amino acids within the active site. VapC is counteracted by the VapB antitoxin [102]. Expression of the *vapB-vapC* genes is autoregulated negatively by the VapB-VapC complex which specifically binds inverted repeat sequences within the *vapBC-1* operator region [103,104]. However, the *vapBC-1* cassette of nontypeable *Haemophilus influenzae* presents notable differences to the typical pattern. First, contrary to other TA systems described to date, the VapC-1 toxin possesses DNA binding activity whereas the VapB-1 antitoxin does not directly interact with DNA. Nevertheless, VapB-1 increases VapC-1 interaction specificity with the operator region [62]. Second, the Fis protein upregulates *vapBC-1* expression during nutrient upshift in *H. influenzae*. The influence of Fis on *vapBC-1* expression is thought to occur indirectly by altering DNA structure in the promoter-operator region [62].

## 8. Type II TAs that Regulate Other Genes or Do Not Display Autoregulation

The *mqsRA* module encoded by *E. coli* displays many features that differ from canonical type II TA systems. First, the gene for the *mqsR* toxin precedes the *mqsA* antitoxin gene, whereas the standard organization comprises an antitoxin gene followed by a toxin gene (Figure 1). This unusual genetic organization has been observed only rarely in other TA systems characterized to date, including the *higBA* [105] and *hicAB* modules [106]. Second, the MqsA antitoxin is well-ordered throughout its entire length and requires zinc ions to maintain its structural stability, properties that are unique among known antitoxins (Figure 2D) [48].

Autoregulation of the *mqsRA* module also is distinctive. The antitoxin repressor, MqsA, undergoes extensive domain rearrangements upon DNA binding and is the only antitoxin known to interact with DNA via its C-terminal domain [48,107]. However, the MqsA N-terminal domain, which binds the MqsR toxin, also makes direct interactions with DNA. It twists and collapses over the DNA and this rotation clamps the DNA thereby enhancing binding [107]. Interestingly, the MqsR toxin does not function as a transcriptional corepressor as in many other TA systems. Instead, MqsR destabilizes the MqsA-DNA complex. This reflects that the binding sites of DNA and MqsR on MqsA partially overlap rendering simultaneous binding of both by MqsA impossible. Thus, MqsR is a transcriptional activator of *mqsRA* expression, not a transcriptional repressor [85].

Distinct from other type II TA systems, the MqsR-MqsA complex also regulates other genomic promoter regions. Specifically, the MqsA antitoxin and the MqsR-MqsA complex regulate the promoters of genes that are important for *E. coli* metabolism, including the *mcbR*, *spy* and *cspD* loci [48,108]. The *mcbR* gene encodes a colanic acid regulator, Spy is a periplasmic chaperone of proteins, and CspD is a stress-induced cold shock protein that is a DNA replication inhibitor. MqsA also directly binds to an MqsRA-like palindrome located within the promoter of the *rpoS* gene and thereby represses transcription of a major regulator of stress, σ^S^ [109]. This lowers the concentration of the internal messenger 3,5-cyclic diguanylic acid (c-di-GMP) which in turn causes increased motility and reduction of biofilm formation, as well as decreased oxidative stress resistance through catalase activity [107]. MsqA also modulates biofilm formation by acting as a transcriptional regulator of the gene that encodes CsgD, a master controller of biofilm formation [110]. The endoribonuclease toxin MqsR is also a global regulator [108,111]. It cleaves specific mRNA mainly at 5’-GCU-3’ sites [112,113] which significantly increases the presence of mRNAs coding for CstA, CspD, RpoS, Dps and HokD proteins that are known to be associated with stress response [108]. Thus, the MqsR-MqsA system controls cell physiology both by its own toxicity as well as through the regulation of other genes.

The chromosomal *mazE-mazF* operon of *Staphylococcus aureus* is transcriptionally linked to the downstream, polycistronic *sigB* operon and is transcribed both as part of the operon and as a shorter transcript. Unlike the homologous genes in *E. coli*, staphylococcal *mazE-mazF* is not subject to autoregulation. Instead, the activity of the *mazE-mazF* promoter is inhibited by the product of the *sigB* gene that codes for the alternative sigma factor, σ^B^. Moreover, the *mazE-mazF* promoter is required for full σ^B^ activity due both to its transcriptional coupling with the *sigB* operon via readthrough of a weak downstream *rho*-independent terminator and to the ability to respond to multiple stresses [114]. On the other hand, the *mazE-mazF* promoter is positively and directly regulated by SarA, a winged-helix, global transcriptional regulator of virulence gene expression in *S. aureus* [115]. This probably occurs by binding of SarA to a region that overlaps the −35 box in the *mazE-mazF* promoter and which exhibits high similarity to the consensus SarA binding site [114]. The intricate transcriptional control of the *mazE-mazF* genes in *S. aureus* highlights an emerging theme in understanding TA activity and function: TA systems frequently form parts of complex gene regulation networks that respond to diverse environmental and physiological signals.

## 9. Information from Toxin-Antitoxin-DNA Costructures

Although a limited number of type II toxin-antitoxin-DNA costructures are available currently, these structures provide valuable insights into the mechanism of DNA binding by antitoxins and how toxins coregulate this activity. For the FitA-FitB complex, a heterotetramer is formed by the binding of two FitB PIN domain toxin monomers to a pair of FitA antitoxin dimers. The FitA antitoxin dimers are thereby tethered to operator DNA. FitA binds DNA via a RHH fold. The toxin monomers do not interact directly with DNA, but promote binding by stabilizing the FitA-FitB heterotetramer (Figure 2C) [47].

The structure of the VapB2-VapC2 TA complex of *Rickettsia felis* with DNA has been determined [49]. As with the FitA-FitB complex, the VapB2-VapC2 structure comprises a tetramer of heterodimers in which four toxin subunits assemble together with four antitoxin subunits (Figure 2E). The VapC2 toxin harbours a PIN domain that forms homodimers homologous to those of FitB. However, unlike the RHH structure of FitA antitoxin, the VapB2 antitoxin dimers contain a swapped-hairpin β-barrel fold, similar to MazE described above. The interaction of VapB2 with DNA is mediated by the β-hairpins. Moreover, the DNA structure is distorted when bound by the TA complex with the VapB2 homodimers interacting with the concave face of the curved DNA [49]. The VapB2-VapC2 complex provides an interesting illustration of how extensive evolutionary shuffling of toxin and antitoxin genes has occurred resulting in considerable diversity in TA combinations [3,71,72].

The HipA toxin possesses a eucaryotic serine/threonine kinase-like fold that structurally is most related to human CDK2/cyclin A kinase [26]. HipB antitoxin cooperatively binds four operator sites (consensus sequence 5’-TATCCN_8_GGATA-3’) located in the *hipBA* promoter region thereby repressing *hipBA* expression. HipA is a co-repressor [116]. HipB is dimeric each subunit comprising a single β strand and four α helices. Two of the helices form a canonical helix-turn-helix motif that mediates DNA major groove contacts. The HipB structure is related to the Cro repressor family of proteins. The protein induces a 70° bend in its operator DNA that assists cooperative binding by aligning the recognition helices for specific binding to successive major grooves [26]. The HipA-HipB structure assembled on DNA consists of dimeric HipB flanked on either side by one HipA monomer. The N-terminal domains of HipA interact with one of the HipB subunits, whereas the HipA C-terminal domains mainly contact the second HipB subunit. These and other interactions trap HipA in an inactive, non-toxic conformation when bound with HipB on DNA. Interestingly, although HipA alone does not bind DNA, a pair of residues in each monomer within the HipA-HipB-DNA structure make phosphate backbone contacts with operator DNA [26].

## 10. New Antibiotics that Interfere with TA Transcriptional Autoregulation to Detonate Toxins Artificially

Bacteria evolve rapidly. Shuttling of mobile genetic elements - plasmids, transposons, bacteriophage and integrons - between bacteria is especially important in promoting genome plasticity and diversification [117,118]. By acquiring antibiotic resistance genes that are frequently located on these elements, bacteria adapt, survive and proliferate in antibiotic containing niches in which sensitive strains perish. The spread of resistance genes on mobile elements in bacteria has been compounded significantly by antibiotic misprescription, by use of antibiotics as growth promoting and prophylactic agents in animals, and by indiscriminate release of antibacterials into the biosphere [119]. Thus, the global surge in antibiotic usage inexorably has selected for bacterial isolates that are resistant not only to single compounds, but in many cases to multiple drugs [120]. As a consequence, certain infections that previously were treatable now have few, if any, therapeutic options [121]. For example, one-third of the world’s population is infected with *Mycobacterium tuberculosis*, the causative agent of tuberculosis, which causes two million deaths globally per annum. The emergence of multidrug resistant strains, then extensively drug resistant isolates, and more recently totally drug resistant strains of *M. tuberculosis* threatens even further devastation in developing countries, as well as the deadly re-emergence of an ancient scourge in the Western world [122]. Similarly, multiresistant strains of Gram-negative *Pseudomonas aeruginosa*, Acinetobacter spp., and Enterobacteriaceae that produce extended-spectrum beta-lactamases are serious hospital-acquired pathogens that are becoming evermore difficult to treat with existing antibiotics, as are Gram-positive species including methicillin-resistant *Staphylcocccus aureus* and vancomycin-resistant enterococci [119,120,123]. Despite the impending crisis, only one new class of antibiotic has come to market in the past 50 years. Moreover, pharmaceutical companies are withdrawing from antimicrobial development in pursuit of more lucrative therapeutics: a perilous gap has opened in the race for novel antibacterials [124].

Ectopic expression of toxin genes induces severe bacterial growth defects, including cell death. Moreover, genes for TA complexes have no known human homologues and are abundant on chromosomes and plasmids of most bacteria, including pathogenic species, which contributes to their attractiveness as potential targets for novel antibacterial agents. There is considerable interest in identifying natural and artificial molecules that destabilize TA complexes to promote toxin activation [125]. A variety of different strategies may be implemented to exploit TA systems to this end. For example, the disruption of the toxin-antitoxin interaction may free the former to induce cell death [126]. Similarly, interference with antitoxin oligomerization will destabilize the TA complex thereby liberating the toxin. In both cases, perturbation of TA complex assembly or organization also is predicted to interfere with correct transcriptional autoregulation. Moreover, compounds that bind the TA promoter region will directly inhibit transcription of TA genes. Replenishment of the labile antitoxin will be prevented thereby releasing the more stable toxin to induce bacterial cell suicide [90]. Similarly, ligands that block antitoxin interaction with the operator site may derepress TA gene expression, imbalance the TA ratio and, again, cause inappropriate toxin release. Persister cells comprise a rare, antibiotic tolerant fraction of bacterial populations. TA loci are implicated in bacterial persistence as the toxin factors reduce metabolism and render cells insenstive to many antibiotics [11]. This phenomenon needs to be considered carefully when assessing the effect of artificial toxin release on bacterial survival.

Studies on disruption of the streptococcal ε-ζ interaction and inhibition of TA complex formation in the anthrax agent of *Bacillus anthracis* suggest that designer peptides may be useful in modulating TA system interactions [127,128,129]. Peptides based on the structure of the ParE toxin have been used as inhibitors of bacterial topoisomerases [130]. Analogously, the Extracellular Death Factor is an endogenous pentapeptide activator of the MazF toxin [60] which indicates the existence of natural ligands that may be exploited to moderate TA complex function. Although these initial studies suggest that peptidomimetics may be potentially powerful in disrupting TA function, designing effective molecules is not straightforward [131]. Developing molecules that alter TA transcriptional regulation directly is an alternative approach for controlled toxin release. Sequence-specific DNA binders already function, for example, in anticancer therapies [132,133,134]. Potential antibacterial drugs targeting TA systems could alter interactions of antitoxins with DNA regulatory sites by a variety of different mechanisms. Drugs that target DNA or change its architecture may compete for antitoxin DNA binding sequences. Synthetic oligonucleotides or other ligands may compete for DNA recognition motifs on the antitoxin. Ligand binding to other regions of the antitoxin may change its ability or sensitivity to interact with regulatory DNA. New weapons are urgently required in the war against infectious disease and antibiotic resistance. The toxin factors in TA complexes have evolved to induce intracellular damage, albeit in a controlled way. By devising strategies that artificially unleash the toxin, including by disrupting proper transcriptional regulation, it may be possible to develop novel antibacterial strategies based on bacterial suicide from within.
